# Vitiligo—Current Treatment Options and Future Perspectives

**DOI:** 10.3390/ijms27146374

**Published:** 2026-07-17

**Authors:** Aleksandra Wojno, Agata Wojno, Anna Karwowska, Milena Chmielewska, Joanna Maj, Magdalena Łyko

**Affiliations:** 1 Student Research Group of Experimental Dermatology, University Centre of General Dermatology and Oncodermatology, Wroclaw Medical University, 50-556 Wrocław, Poland; 2 University Centre of General Dermatology and Oncodermatology, Wroclaw Medical University, 50-556 Wrocław, Poland

**Keywords:** vitiligo, JAK inhibitors, ruxolitinib, phototherapy, calcineurin inhibitors, repigmentation, melanocyte transplantation, combination therapy

## Abstract

Vitiligo is a chronic autoimmune disease characterized by the loss of melanocytes, resulting in the appearance of depigmented patches on the skin. The aim of the present review is to discuss current therapeutic methods and to outline promising directions for the treatment of this dermatosis. The review describes pathogenetic mechanisms, including the role of oxidative stress, immune dysregulation, and the JAK/STAT pathways, which constitute the foundation of contemporary therapeutic interventions. The efficacy and safety of topical treatments (corticosteroids, calcineurin inhibitors, JAK inhibitors), phototherapy (NB-UVB, PUVA, excimer laser), combination therapies, and surgical methods such as cellular and tissue grafts are discussed. Data indicate that the combination of phototherapy with topical agents enhances repigmentation efficacy, and JAK inhibitors represent promising drugs in targeted therapy. Particular value is attributed to preparations modulating the immune response and to melanocyte transplantation techniques, which emerge as highly effective and forward-looking treatment options for vitiligo. Nevertheless, effective management of vitiligo requires a multidirectional approach combining pharmacological, immunomodulatory, and regenerative interventions.

## 1. Introduction

Vitiligo, also known as acquired depigmentation disorder, is a chronic autoimmune disease characterized by the loss of melanocytes, leading to the formation of depigmented patches on the skin [1]. The prevalence of vitiligo varies across different geographic regions and ethnic groups. Worldwide, the prevalence is generally estimated at approximately 0.5–2%, with reported figures varying from below 0.1% to over 2% depending on the population studied and the diagnostic methodology used [2]. It is estimated that around 20–30% of vitiligo cases occur in individuals with a positive family history, suggesting a possible hereditary component [2]. The disease often manifests in childhood, with many cases presenting initial lesions before the age of 20. A higher prevalence is observed among females [2]. To provide a more precise classification of its clinical heterogeneity, vitiligo is categorized into several subtypes: non-segmental, segmental, and unclassified vitiligo [3].

Non-segmental vitiligo (NSV) is the most common form of the disease, accounting for approximately 85–90% of all cases. It is characterized by the symmetrical distribution of depigmented patches, which may affect the skin of the face, hands, and legs, as well as friction-prone areas such as the elbows and knees. This form of vitiligo is progressive, meaning that the lesions can expand to other areas of the body, leading to extensive pigment loss [3]. Autoimmune mechanisms play a crucial role in its pathogenesis, along with an increased susceptibility to other immune-related disorders, such as type 1 diabetes and thyroid diseases [1].

Segmental vitiligo (SV) is a less common form of the disease, accounting for approximately 5–30% of all cases. Unlike NSV, it affects only one side of the body and is characterized by the unilateral distribution of depigmented patches. The lesions typically involve a specific area innervated by a single dermatome. This type of vitiligo generally follows a relatively stable course, meaning that after an initial phase of progression, the disease often ceases to advance [3]. However, in some patients, mixed forms of vitiligo may occur, in which SV coexists with NSV, referred to as the mixed type [3].

Unclassified vitiligo includes cases that do not fit into the two main categories. Among them is universal vitiligo, an extreme form of NSV, characterized by near-complete depigmentation of the skin and hair. This condition involves widespread loss of melanocytes across the entire body, leading to significant esthetic and psychological consequences for affected individuals. Another subtype, acrofacial vitiligo, primarily affects the extremities and the face, particularly around the eyes and mouth. In contrast, mucosal vitiligo is confined to the mucous membranes of the oral cavity, nose, and genital areas [3]. Detailed information is described in Table 1.

This narrative review was based on a targeted literature search of PubMed, Embase, and Web of Science databases, covering the period from January 2016 to March 2026. Given the mechanistic scope of this review, seminal earlier studies relevant to disease pathophysiology and phototherapy mechanisms were additionally included where appropriate, so that this period was applied flexibly rather than as a strict cut-off. The following search terms were used in various combinations: “vitiligo” AND (“treatment” OR “therapy” OR “JAK inhibitor” OR “phototherapy” OR “calcineurin inhibitor” OR “surgical” OR “melanocyte transplantation” OR “combination therapy” OR “emerging therapy”). Inclusion criteria encompassed original research articles, randomized controlled trials, systematic reviews, meta-analyses, and narrative reviews published in peer-reviewed journals. Reference lists of included articles were manually screened for additional relevant publications. This review was not registered in PROSPERO, as it follows a narrative rather than a systematic methodology.

## 2. Risk Factors and Pathogenesis of Vitiligo

The pathogenesis of vitiligo is multifactorial and involves immune mechanisms, oxidative stress, genetic susceptibility, and neurogenic factors. In the autoimmune model, CD8+ T lymphocytes target melanocytes, inducing their apoptosis through the activation of interferon-gamma (IFN-γ) and the CXCL9/10 chemokine pathway, leading to chronic autoaggression [10]. Melanocyte-specific cytotoxic CD8+ T cells are key effector cells in vitiligo, as they accumulate at the dermal–epidermal junction and perilesional margins, recognize melanocyte antigens, and directly contribute to melanocyte apoptosis/destruction [1,10,11]. Their production of IFN-γ activates the CXCL9/CXCL10–CXCR3 and JAK/STAT pathways, promoting further recruitment of autoreactive CD8+ T cells, whereas CD8+ tissue-resident memory cells, supported by IL-15 signaling, are linked to persistence and relapse of vitiligo lesions [1,6,10,12].

**Figure 1:**
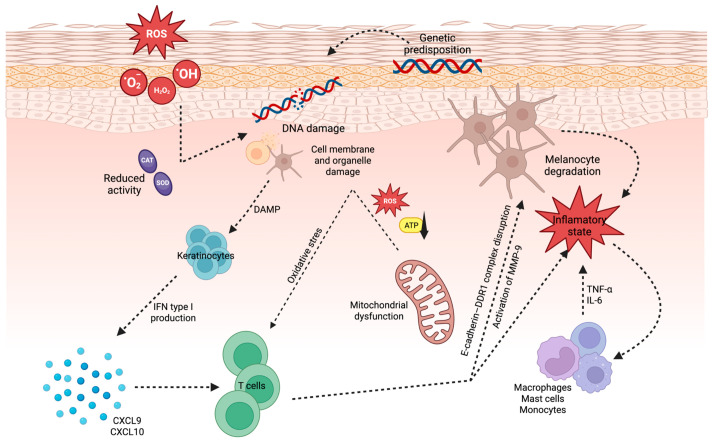
In the pathogenesis of vitiligo, redox imbalance initiates a cascade of events that ultimately leads to the loss of melanocytes. Excess reactive oxygen species (ROS), particularly hydrogen peroxide, combined with decreased activity of antioxidant enzymes, such as catalase and superoxide dismutase, result in DNA damage, disruption of cell membranes and organelle, and activation of apoptotic pathways. Damage-associated molecular patterns (DAMPs) released from injured melanocytes stimulate dendritic cells and keratinocytes to secrete type I interferons, thereby inducing the expression of chemokines CXCL9 and CXCL10. This chemokine gradient recruits melanocyte-specific CD8+ T cells to the skin, where they recognize melanocytic antigens and mediate cytotoxic destruction of these cells [10]. Created in BioRender. Łyko, M. (2026): https://BioRender.com/zp67zhi. Arrow: decreased ATP production.

Oxidative stress plays a crucial role in disease initiation—melanocytes in vitiligo patients are more susceptible to reactive oxygen species (ROS), resulting in DNA damage, membrane destabilization, mitochondrial dysfunction, and ultimately their elimination. Elevated levels of hydrogen peroxide (H_2_O_2_) and deficiencies in antioxidant enzymes, such as catalase and superoxide dismutase, further highlight the role of redox dysregulation in pathogenesis [13] (Figure 1). Excessive ROS can damage mitochondrial DNA, lipids and proteins, impair the electron transport chain and ATP production, and thereby further increase mitochondrial ROS generation, creating a self-amplifying oxidative–mitochondrial loop in melanocytes [1,5,9]. This mitochondrial impairment may promote melanocyte apoptosis through cytochrome c release and caspase activation, while SIRT3-related mitochondrial dysfunction has also been linked to oxidative-stress-induced melanocyte death [14,15,16].

Ferroptosis should also be included among oxidative-stress-related mechanisms of melanocyte loss in vitiligo. This iron-dependent form of regulated cell death is driven by lipid peroxidation and has been linked to cystine/glutamate antiporter dysfunction, glutathione depletion, GPX4 inactivation, mitochondrial abnormalities, iron accumulation, and altered expression of ferroptosis-related markers such as ferritin, transferrin receptor 1 and ACSL4 [13,14,15,16]. Although its precise contribution to vitiligo remains incompletely defined, current evidence suggests that ferroptosis may represent a relevant pathogenic pathway as well as a potential biomarker and therapeutic target [13,15,16].

Additionally, oxidative stress/ROS and impaired Keap1–Nrf2–ARE signaling reduce melanocyte antioxidant capacity and promote DAMP/HSP70 release, dendritic-cell activation and CD8+ T cell recruitment [14,16,17]. The IFN-γ–CXCL9/CXCL10–CXCR3 axis, mediated through JAK1/JAK2–STAT1 signaling, amplifies inflammation by inducing keratinocyte chemokine production and further recruitment of autoreactive CD8+ T cells [6,10,11,14]. IL-15/JAK–STAT signaling supports CD8+ tissue-resident memory T cells, which produce IFN-γ, TNF-α, CXCL9 and CXCL10 and contribute to lesion persistence and relapse [1,12,16]. Additional melanocyte-intrinsic pathways include cAMP–PKA–CREB/MITF and MAPK signaling, which regulate melanogenesis, as well as DKK1/Wnt/β-catenin and E-cadherin/DDR1/MMP-9-related mechanisms, which may impair melanocyte function, adhesion and survival [12,14,16].

Impairment of tetrahydrobiopterin (BH4)-dependent hydroxylation of phenylalanine to tyrosine represents an early stage of melanogenesis and constitutes an important metabolic mechanism. In this process, tyrosine is subsequently converted by tyrosinase to DOPA and further melanin precursors. In vitiligo, a three- to five-fold excess of BH4 has been reported, which may inhibit tyrosinase, disrupt the BH4 regeneration cycle, and enhance H_2_O_2_ production, thereby establishing a link between defective melanogenesis and oxidative stress. In addition, oxidation and UV-induced phototransformation of BH4 may lead to the formation of pterin products and dihydropterin dimers, which have been associated with the pathogenesis of vitiligo and with a potential mechanism of action of 308/311 nm UVB phototherapy [18,19,20,21,22].

Amino acid-related changes are also important biomarkers of vitiligo. Phenylalanine and tyrosine are directly linked to the early stages of melanogenesis, as BH4/H4Bip-dependent phenylalanine hydroxylase converts phenylalanine to tyrosine, which is subsequently metabolized by tyrosinase to DOPA and further melanin precursors. Disturbances in the BH4/H4Bip–phenylalanine–tyrosine axis may so reflect impaired pigment biosynthesis and oxidative stress [18,19,20]. Cysteine and glutamic acid/glutamate are linked to redox homeostasis and ferroptosis, because the cystine/glutamate antiporter system Xc^−^ regulates cysteine availability for glutathione synthesis, while GSH depletion and GPX4 inactivation promote lipid peroxidation and melanocyte death [13,16]. These amino acids should be presented as potential adjunctive biomarkers, especially because omics-based biomarker studies in vitiligo still require broader validation and methodological standardization [23]. Beyond amino acids, other low-molecular-weight candidates discussed in the literature include biopterin and oxidized pterins such as pterin-6-carboxylic acid [24], whereas arachidonic acid and circulating microRNAs remain preliminary candidate markers that require further validation [21].

Genetic predisposition is also a key factor, as demonstrated by genome-wide association studies (GWASs), which have identified polymorphisms in genes associated with the immune system and melanocyte metabolism, including major histocompatibility complex (MHC) class I genes and pro-inflammatory cytokines [10,14,15]. Familial and epidemiological data further support this genetic contribution, although the risk is not absolute: first-degree relatives have an increased risk, and family history was reported in 15.9% of patients in a North Indian cohort and in 29.6% of patients in a Brazilian cohort, while current data indicate a polygenic, multifactorial model rather than a single-gene inheritance pattern [6,7,10,14].

Additionally, neurogenic factors may contribute to disease progression, as suggested by increased expression of neuropeptides such as substance P and neurokinin A, which modulate inflammatory responses and influence melanocyte survival [14].

Concurrently, mitochondrial dysfunction occurs, including impairments in respiratory chain complex I and a reduction in cardiolipin levels. These alterations lead to increased mitochondrial ROS production and ATP depletion, further exacerbating oxidative stress and promoting premature melanocyte senescence. Senescent melanocytes acquire a senescence-associated secretory phenotype (SASP), releasing pro-inflammatory cytokines that amplify the local immune response and facilitate further lymphocyte recruitment [15]. The presence of pro-inflammatory cytokines, particularly interferon-gamma (IFN-γ), activates the JAK/STAT1 signaling pathway in keratinocytes and melanocytes, inducing the expression of interferon-stimulated genes, including chemokines and matrix metalloproteinases. Activation of MMP-9 and disassembly of the E-cadherin-DDR1 complex result in melanocyte detachment from the basement membrane and their elimination. Simultaneously, IFN-γ maintains CXCL10 expression, perpetuating local inflammation [11]. Genetic susceptibility, oxidative stress and cytokine signaling converge in vitiligo: HLA/MHC and immune-regulatory variants may facilitate melanocyte antigen presentation, while IFN-γ/JAK/STAT1-driven CXCL9/CXCL10 production recruits CXCR3+ CD8+ T cells and sustains local autoimmune inflammation [1,6,11,14].

Chronic inflammation fosters the long-term presence of CD8+ T cells in the skin, including resident memory T cells (Trm), which express CD69 and CD103 and are capable of rapidly producing IFN-γ and CXCL10 upon local stimulation. The presence of Trm cells at the periphery of lesions contributes to vitiligo relapse even after clinical remission and discontinuation of therapy [10].

Simultaneously, the balance between effector and regulatory T cell populations is disrupted. A reduction in both the number and function of regulatory T cells (Tregs) impairs suppression of the cytotoxic immune response, contributing to its persistence. Local immune and keratinocyte metabolism is also altered, with reprogramming of glycolysis and fatty acid oxidation pathways that support effector T cell survival and intensify cytokine production [16]. Active vitiligo lesions also show increased activity of natural killer (NK) cells and innate lymphoid cells (ILCs), which secrete IFN-γ and modulate CXCR3B receptor expression on melanocytes, increasing their vulnerability to chemokine-induced apoptosis. The cooperation between innate and adaptive immune responses results in the breakdown of melanocyte immune privilege and their full immunogenicity within the inflammatory environment [10].

Genetic predisposition is a critical factor enabling the initiation and progression of the cytotoxic response. Polymorphisms in HLA class I genes, as well as genes encoding cytokines, JAK/STAT pathway proteins, and melanocyte-associated proteins such as TYR and MC1R, increase the risk of autoimmune targeting of melanocytes [14].

Affected skin areas also exhibit local activation of macrophages, mast cells, and monocytes, which secrete IL-6, TNF-α, and other mediators, creating a microenvironment conducive to sustaining inflammation. The metabolism of keratinocytes and immune cells in these areas displays characteristics of energy deficiency and chronic oxidative stress, impairing regenerative capacity and antigen tolerance. Ultimately, this sequence leads to the irreversible depletion of melanocytes from the epidermis and the development of depigmented lesions. This process is sustained by persistent inflammatory cytokine signaling, oxidative stress, the presence of Trm cells, insufficient immune regulation, and metabolic dysfunction—factors that collectively render vitiligo a chronic, relapsing, and difficult-to-treat autoimmune condition [12,13,17].

## 3. Diagnosis of Vitiligo

The diagnosis of vitiligo is primarily based on clinical evaluation, with Wood’s lamp being a fundamental diagnostic tool. This device emits ultraviolet light, enhancing the visibility of depigmented skin areas, particularly in patients with lighter skin tones [3]. In cases requiring histological confirmation, a skin biopsy is performed, revealing the absence of melanocytes in the basal layer of the epidermis [3]. Dermoscopy is also utilized to assess subtle pigmentary changes, while more advanced cases may require reflectance confocal microscopy and high-resolution ultrasound to analyze skin structure [3]. Additionally, proteomic studies are gaining increasing significance in identifying disease biomarkers and potential therapeutic targets, highlighting alterations in protein expression related to immune processes and oxidative stress [23].

## 4. Treatment

The treatment of vitiligo remains a clinical challenge, as therapeutic response is variable and complete repigmentation is rarely achieved. Management strategies are guided by the extent, location, activity, and duration of the disease, as well as the patient’s age and preferences. The therapeutic approach aims to halt disease progression, stimulate repigmentation, and improve cosmetic outcomes.

This chapter provides a comprehensive overview of current therapeutic options for vitiligo focusing on topical treatments—such as corticosteroids, calcineurin inhibitors, and Janus kinase (JAK) inhibitors, phototherapy modalities including narrowband ultraviolet B (NB-UVB) and excimer laser, and combination regimens that enhance efficacy. Surgical interventions, though typically reserved for stable and refractory cases, will also be addressed.

### 4.1. Topical Treatment

#### 4.1.1. Topical Corticosteroids

Topical corticosteroids (TCSs) are used in various dermatological conditions such as psoriasis, eczema, atopic dermatitis, and discoid lupus erythematosus, as well as in cases of localized vitiligo [8].

In the treatment of vitiligo, first-line corticosteroids include highly potent corticosteroids such as betamethasone valerate and clobetasol propionate. The therapy involves applying high-potency TCS daily to small areas of the body, while in more sensitive areas such as the face, neck, or skin folds, the use of calcineurin inhibitors or lower-potency corticosteroids is recommended. This is due to the increased risk of adverse effects in these regions [8].

The side effects of TCS primarily include skin atrophy, which is more frequently observed with the use of class 4 corticosteroids compared to class 3. Other side effects include stretch marks, excessive hair growth, acneiform eruptions and telangiectasias [25].

The treatment regimen includes the daily use of TCS for a period of three months. Intermittent therapy for up to six months is acceptable. If no changes are observed after three to four months, the treatment should be discontinued [26]. After applying TCS, the expected change is repigmentation in depigmented areas. If repigmentation does not occur, therapy should be switched to calcineurin inhibitors or, in the case of further disease progression, to phototherapy [27].

#### 4.1.2. Topical Calcineurin Inhibitors

Topical calcineurin inhibitors (TCIs) are used as an induction therapy in limited vitiligo on face, neck and body folds such as axillary and inguinal regions [17]. Tacrolimus and pimecrolimus are used off-label for treating vitiligo [28,29]. TCIs can be used as monotherapy and as combination therapy [30]. In a randomized double-blind trial involving pediatric patients with vitiligo, topical 0.1% tacrolimus demonstrated comparable efficacy to 0.05% clobetasol propionate, achieving a mean repigmentation of 41.3% versus 49.3%, respectively, but without steroid-associated side effects such as skin atrophy or telangiectasia—highlighting its value for long-term use, especially on sensitive areas like the face or eyelids [31]. While oral calcineurin inhibitors are associated with elevated risk of malignancy, such as lymphomas or skin cancer, a study showed no correlation of these malignancies in patients treated with topical calcineurin inhibitors and phototherapy or both [32]. Side effects of TCIs that can be present during treatment are: burning, irritation, stinging and pruritus [29]. Combined treatment of TCIs and phototherapy did not result in an intensified photocarcinogenesis and a study on mice reported that TCIs prevent UV light’s DNA photodamage. Additionally, a mouse model showed that TCIs can inhibit the induction of skin tumors [33,34]. Applying TCIs twice a week intermittently on facial vitiligo can prevent its recurrence and can be used as maintenance therapy [35].

#### 4.1.3. Topical Ruxolitinib

Topical ruxolitinib is the first and only currently FDA- and EMA-approved JAK inhibitor for the treatment of vitiligo in adults and children over the age of 12 [3,36]. Ruxolitinib is a selective JAK1 and JAK2 inhibitor. The inhibition of JAK1 and JAK2 inhibits the JAK-STAT pathway and leads to a lowering of the level of chemokines such as CXCL10 thereby reducing the size of skin depigmentation [37]. The use of this 1.5% cream can result in facial repigmentation even by week 4 [36]. In two phase 3 trials with a total of 674 patients, Rosmarin et al. reported that ruxolitinib used twice daily for 52 weeks resulted in more promising results in repigmentation than the vehicle control, with acne and pruritus at the application site as adverse events. Patient-reported outcomes were favorable throughout 24 weeks of double-blinded therapy [38]. More JAK inhibitors are currently being investigated for the treatment of vitiligo [39]. A summary of clinical studies on topical therapies for vitiligo is presented in Table 2.

### 4.2. Phototherapy

Phototherapy remains one of the cornerstone treatment modalities for vitiligo, particularly for generalized or widespread disease. Its primary mechanism involves immunomodulation and stimulation of melanocyte proliferation and migration from hair follicles or perilesional skin. The following sections describe the most commonly used phototherapeutic approaches: narrowband UVB (nb-UVB), psoralen plus UVA (PUVA), and excimer laser therapy [27].

#### 4.2.1. NB-UVB

Recent evidence suggests that, beyond classical immunomodulatory effects, phototherapeutic efficacy in vitiligo may also involve emerging photobiochemical mechanisms, including two-photon absorption phenomena, which could influence pterin-related pathways and melanogenesis.

Narrowband UVB (311–313 nm) is currently considered the first-line phototherapeutic modality for non-segmental vitiligo. It is preferred due to its favorable safety profile, efficacy, and ease of use. NB-UVB induces local immunosuppression, downregulates pro-inflammatory cytokines, and promotes melanocyte proliferation and migration. It also enhances melanin synthesis in melanocytes. Treatment is typically administered 2–3 times weekly over several months. Visible repigmentation often begins after 2–3 months, with continued improvement seen over 6–12 months. The face and trunk respond best, while acral and bony areas tend to be more resistant. NB-UVB has demonstrated superior efficacy compared to PUVA in multiple studies. Side effects are generally limited to mild erythema, pruritus, and xerosis. Although long-term phototherapy raises concerns about a potential increased risk of skin cancer, current data do not indicate an increased overall risk of cancer in patients with vitiligo. Furthermore, a recent systematic review and meta-analysis suggests a possible protective effect against some skin cancers, which may be related to the increased immune surveillance and chronic oxidative stress and inflammation associated with vitiligo. Further high-quality studies are needed to clarify these associations [42].

It should be noted that NB-UVB remains an effective monotherapy for vitiligo. Current evidence indicates that combining NB-UVB with topical calcineurin inhibitors or vitamin D analogs does not significantly enhance overall repigmentation outcomes [43]. Studies have reported inconsistent results regarding the correlation between vitiligo and vitamin D levels. In vitro studies have demonstrated that vitamin D3 increases tyrosinase activity and melanogenesis. Vitamin D analogs can improve repigmentation in patients with vitiligo. Furthermore, vitamin D has an immunomodulatory role by suppressing the expression of crucial proinflammatory cytokines. Various cytokines involved in vitiligo pathogenesis are regulated by vitamin D or its analogs [44].

However, combination therapy of NB-UVB with topical calcineurin inhibitors appears to offer superior efficacy for lesions on the face and neck, suggesting a site-specific benefit in these cosmetically sensitive areas [43].

#### 4.2.2. PUVA

PUVA therapy involves the administration of a photosensitizing agent (typically 8-methoxypsoralen or 5-methoxypsoralen) followed by exposure to UVA radiation (320–400 nm). PUVA induces melanocyte activation and proliferation, modulates cytokine profiles, and exerts immunosuppressive effects on T cell-mediated inflammation. PUVA can be delivered systemically (oral psoralen) or topically (bath or cream psoralen), followed by UVA exposure. Systemic PUVA is used less frequently today due to its side effect profile. Treatment is generally administered 2–3 times per week. PUVA is effective, particularly in segmental and acral vitiligo, but its use has declined due to higher risks of phototoxicity, gastrointestinal intolerance, and potential long-term carcinogenicity. Eye protection is mandatory due to the risk of lenticular damage [45].

NB-UVB phototherapy has demonstrated greater efficacy than PUVA in inducing repigmentation, especially in cases of unstable and widespread vitiligo, and is more likely to result in uniform and cosmetically acceptable pigmentation. Consequently, NB-UVB is generally regarded as the preferred phototherapeutic modality for vitiligo. However, PUVA may still be considered in selected patients, particularly when NB-UVB fails to elicit an adequate response [8,25,26,43].

#### 4.2.3. Excimer Laser

The 308 nm excimer laser represents a significant tool in the treatment of localized vitiligo lesions. Emitting light with a wavelength similar to that of NB-UVB, it induces T cell apoptosis and stimulates both the proliferation and migration of melanocytes. This laser is particularly effective in treating small depigmented areas and is characterized by a favorable safety profile. Randomized clinical trials have shown that excimer laser therapy combined with a topical calcineurin inhibitor, such as tacrolimus, significantly increases the proportion of patients achieving ≥75% repigmentation compared to laser monotherapy [46].

Side effects are usually mild and include erythema and blistering. In addition to inducing T cell apoptosis and stimulating melanocyte proliferation, the 308 nm excimer laser may also exert its effects through photochemical mechanisms related to pterin metabolism. Experimental studies indicate that irradiation at this wavelength promotes the formation of chemically intact dihydropterin dimers, thereby limiting the accumulation of oxidized pterins, which contribute to increased oxidative stress in melanocytes of patients with vitiligo [24,47,48]. A summary of phototherapy studies and findings in vitiligo treatment is presented in Table 3.

### 4.3. Combined Therapies

Monotherapy in vitiligo often yields suboptimal or slow repigmentation, particularly in resistant or extensive disease. As a result, combined therapeutic strategies have gained increasing attention for their potential to enhance efficacy through synergistic mechanisms. The rationale for combination therapy lies in targeting multiple pathogenic pathways simultaneously—such as immunosuppression, melanocyte stimulation, and oxidative stress reduction—to improve clinical outcomes while minimizing adverse effects.

This chapter explores the evidence and clinical utility of various combination therapies in vitiligo, focusing on efficacy, safety profiles, and practical considerations for patient selection and treatment planning.

Furthermore, an additional important combination therapy strategy for vitiligo is the use of topical pseudocatalase in combination with narrowband UVB phototherapy (NB-UVB). This was developed by Schallreuter and colleagues and is based on the hypothesis that oxidative stress plays a key role in melanocyte destruction. Pseudocatalase is designed to degrade hydrogen peroxide (H_2_O_2_) in the epidermis, restoring redox balance and improving melanocyte function. When combined with UVB irradiation, this strategy may promote repigmentation by reducing oxidative damage and promoting melanocyte regeneration, but clinical responses are variable. Further studies are needed [49,50].

In Europe and the United States patients with NSV are most commonly treated with TCIs and phototherapy [4]. NSV is linked to autoimmune dysfunction. As immunosuppressants, TCIs were found to be more fitting for treating NSV rather than segmental vitiligo [51]. Apart from being immunosuppressants, TCIs enhance melanocyte induction [39]. Tacrolimus 0.1% was found to be efficacious for treating facial vitiligo in adult patients when applied twice a day. For more successful facial repigmentation TCI therapy should be paired with natural UV light therapy [40]. Body parts that are usually covered from sunlight such as the trunk and extremities showed a lower treatment response to TCI monotherapy than body parts exposed to sunlight [40].

Combining NB-UVB with tacrolimus 0.1% ointment or calcipotriol/betamethasone dipropionate cream leads to a similar efficacy [52].

Bakr et al. showed that in stable NSV combining fractional CO_2_ laser with NB-UVB results in a superior response than combining fractional CO_2_ laser with topical tacrolimus or topical calcipotriol [43].

The efficacy of oral mini-pulse therapy in comparison to other treatments is inconclusive with more clinical trials utilizing validated scales needed to determine it [38].

A combination therapy of pulse steroid (oral mini-pulse or high-dose pulse therapy) for three months and methotrexate (MTX), added after two months of steroid therapy, resulted in a lowering of Vitiligo Extent Score (VES) after six months of treatment which was later verified after 12 months. Here patients who underwent phototherapy experienced a faster repigmentation. A study reported a larger decrease in vitiligo extension after three-month-long combined treatment of oral MTX and oral mini-pulse dexamethasone than after either substance alone. Additionally, intralesional pigmentation occurrence was elevated after the combined treatment and after oral MTX therapy alone and reduced after the oral mini-pulse dexamethasone therapy alone. Furthermore, oral MTX combined with oral mini-pulse dexamethasone is considered a successful treatment for unstable vitiligo with minor side effects [53].

A study reported that the combined treatment of oral mini-pulse and NB-UVB leads to better results than NB-UVB therapy with both excelling oral mini-pulse therapy alone for treating patients with stable vitiligo [54].

One of the combination therapies that can be used in people struggling with vitiligo is the combination of tacrolimus with NB-UVB laser therapy or with an excimer laser. The conducted studies have concluded that such a combination of therapies increases the effectiveness of repigmentation [55].

A meta-analysis found that the combination of NB-UVB with tacrolimus induced greater melanin growth than either phototherapy or topical medications alone. These effects were observed in patients with lesions particularly visible on the face and proximal extremities [55].

Moreover, a multicenter cohort study conducted in Korea involving 25,694 patients with vitiligo provides important information on potential side effects. Based on this study, it was concluded that calcineurin inhibitors combined with NB-UVB phototherapy do not increase the long-term risk of developing lymphoma or skin cancers [32].

In one study, the efficacy of treating patients with nonsegmental vitiligo with phototherapy and JAK inhibitors was assessed. After the use of NB-UVB and baricitinib, an alternative to tofacitinib, Mumford et al. reported that the patient achieved complete repigmentation after 8 months of treatment [56].

Another example of a combination therapy was presented by Li et al., who described two patients with vitiligo treated with baricitinib and NB-UVB therapy, achieving over 75% repigmentation. These findings illustrate the promising potential of the combined treatment approach. Side effects included skin itching and erythema, which is an important consideration for further evaluation of the efficacy and safety profiles of baricitinib in combination with NB-UVB for vitiligo [57].

TCIs, tacrolimus and pimecrolimus, exhibited superior effects compared to placebo in treating vitiligo with phototherapy [51]. It should be noted that TCIs are considered to be less successful than TCS for treating extrafacial vitiligo [58]. Alshiyab et al. reported that combined treatment of 0.1% tacrolimus ointment with NB-UVB phototherapy resulted in similar efficacy to calcipotriol/bethamethasone dipropionate cream combined with NB-UVB after 3 and 6 months [59]. The summary of combined therapies studies and findings in vitiligo treatment are presented in Table 4.

### 4.4. Surgical Treatment

Surgical intervention is a therapeutic option for patients with stable vitiligo who have failed to respond to medical and phototherapeutic treatments. It is primarily indicated in cases of segmental or focal vitiligo with no evidence of disease progression for at least 6–12 months. Surgical methods aim to restore pigmentation by transplanting functional melanocytes to depigmented areas, either through tissue or cellular grafting techniques.

While not appropriate for all patients, surgical treatments can offer significant and often permanent repigmentation, particularly when performed under strict criteria and in appropriately selected candidates. This section will discuss the main surgical modalities, including punch grafting, split-thickness skin grafting, suction blister grafting, and cellular techniques such as melanocyte–keratinocyte transplantation.

#### 4.4.1. Tissue Grafts

Currently, there are several methods used in the treatment of vitiligo. These include surgical methods such as suction blister skin grafting [61]. This technique involves transplanting the epidermis using a suction device that creates negative pressure. This leads to the formation of blisters, causing the separation of the basal layer of the epidermis from the basement membrane of the dermis. The blister roof is then placed on the depigmented skin area, which has been stripped of its epidermis. Ecchymosis is present in the dermis, but no bleeding occurs.

In the past, this method was used in research on blistering diseases. Later, the technique was adapted for skin grafting to transfer melanocytes in patients with depigmented skin lesions [61]. Another method is the mini/micro-punch graft (MPG) transplant; it involves harvesting microscopic skin fragments from the donor and then placing them into appropriately prepared openings in the recipient’s skin [62].

These grafts are carefully secured with a dressing, which is changed after 48 h and then again 7 days after the procedure. Repigmentation can be observed after 2–3 weeks, and individual grafts should merge into a single area within 4–6 months. This method is simple and does not require specialized equipment; however, it cannot be used to treat large areas of skin. MPG has its drawbacks, as it carries the risk of scarring and keloid formation. Side effects may include textural changes and pigmentary disturbances, such as the “cobblestoning” effect [62].

For STSG (split-thickness skin grafts), skin of the desired thickness is harvested using a dermatome and obtained as a thin layer [61].

The graft is then placed on the specially prepared recipient site. The preferred areas for skin harvesting are the flat surface of the thigh, the lower back, or the buttock area. This allows for obtaining a graft of uniform thickness [61].

#### 4.4.2. Cellular Grafts

Surgical procedures can be performed on patients with stable vitiligo. Stable vitiligo is characterized by the lack of new lesions or absence of growth in present lesions. To start surgical treatment the disease should be stable for around 12 months. Cellular grafts usually result in less side effects than in tissue grafts and require a 1:10 donor-to-recipient ratio while tissue grafts are done with a 1:1 donor-to-recipient ratio [29]. Cellular grafts are largely divided into two groups: cultured melanocyte/keratinocyte transplantation and non-cultured epidermal cell suspension (NCES [61,62]). NCES transplantation is the most common cellular graft. This method can be used to treat extensive areas, with a donor-to-recipient ratio reaching up to 1:10. Yet NCES transplantation takes a lot of time as graft processing and the procedure itself are both prolonged. UV-B phototherapy after NCES can amplify repigmentation [63]. Chandela et al. reported a 67% level of repigmentation after NCES by six months with low complication at the donor site. The graft was acquired in a 1:10 ratio from a thigh using Silver’s knife. In this study 69.23% of patients had vitiligo vulgaris and 20.51% focal vitiligo with the stability period varying between 1 and 10 years as NCES is suitable for patients with stable vitiligo. Just 3 out of 39 patients had complications—mild to moderate hyperpigmentation at the donor site [63]. The melanocyte–keratinocyte transplantation procedure (MKTP) is an NCES technique, which can lead to repigmentation of lesions up to 50–100%. Shahbazi et al. reported that autologous MKTP in patients with refractory stable vitiligo is an effective procedure with repigmentation beginning at 4 weeks after transplanting non-cultured melanocytes/keratinocytes [64]. Adverse effects of MKTP include a perigraft halo [65]. MKTP is rather a quick, uncomplicated and low-cost procedure with no need for highly skilled professionals and advanced laboratory equipment [65]. A summary of surgical treatment studies and findings in vitiligo treatment is presented in Table 5.

### 4.5. Emerging Therapies

Emerging treatment approaches for vitiligo include molecularly targeted therapies and advanced repigmentation techniques. Among significant advancements is the application of Janus kinase (JAK) inhibitors, which, due to their specific mechanism of action, have generated considerable clinical interest [11,38]. Beyond the already-approved topical ruxolitinib, the oral JAK inhibitor ritlecitinib, which targets kinases of the JAK3/TEC family, is currently under clinical investigation as a potential therapy for active forms of vitiligo [66,67]. Ritlecitinib has shown significant improvement in the Facial Vitiligo Area Scoring Index (F-VASI) among patients with active NSV. Patients administered 50 mg of ritlecitinib daily demonstrated significant reductions in depigmentation compared to placebo at week 24, with continued improvement observed up to week 48. Moreover, skin type may serve as an important prognostic factor influencing the rate of therapeutic response [68].

A promising therapeutic option is the use of prostaglandin analogs, specifically prostaglandin F2 alpha (PGF2α) and prostaglandin E2 (PGE2), whose efficacy in combination with narrowband ultraviolet B phototherapy (NB-UVB) has been confirmed in clinical trials. Subcutaneous injections of these substances significantly accelerate the repigmentation process, providing an economically accessible and safe adjunctive therapy for stable vitiligo lesions [69].

It is also important to emphasize the effectiveness of autologous melanocyte and keratinocyte transplantation in the treatment of vitiligo. This procedure involves obtaining a thin skin graft, typically from the buttock or thigh area, followed by incubation of the sample in an enzymatic solution, which allows separation of the epidermis containing melanocytes from the dermis, the latter being discarded. Once a cell suspension rich in melanocytes is obtained, it is applied to the affected skin surface that has been previously prepared by dermabrasion [70]. The procedure demonstrates good efficacy in treating lesions both on the face and in other anatomical locations, although slightly superior outcomes have been observed for areas outside the face. The incidence of adverse effects, such as keloids or peripheral hypopigmentation, remains low [71].

An important area of research also remains the application of afamelanotide, a synthetic analog of alpha-melanocyte-stimulating hormone (α-MSH), which exerts its effects by stimulating melanocyte proliferation and melanogenesis. Preliminary research findings indicate that afamelanotide may effectively support repigmentation, particularly when combined with phototherapy or topical medications [72,73].

Oral glucocorticosteroids, particularly when administered in a mini-pulse regimen (OMP), also play a crucial role in the management of active vitiligo. In a study involving 40 patients, dexamethasone or betamethasone was administered at a dose of 5 mg on two consecutive days per week over a two-month period. This approach led to disease stabilization in 89% of participants and induced repigmentation in 80% of them. The treatment was well tolerated, with only mild and transient adverse effects, such as general fatigue and weight gain [72].

An alternative to corticosteroids includes immunosuppressive agents such as cyclosporine, azathioprine, and methotrexate. Although used infrequently, cyclosporine has been employed in cases of generalized, active vitiligo. It has demonstrated efficacy in stabilizing depigmented lesions and promoting partial repigmentation without severe side effects [74]. Azathioprine was evaluated in a study involving patients with chronic, widespread vitiligo, where it effectively reduced disease activity. However, comprehensive data on its long-term efficacy and safety remain limited [75]. Methotrexate, a folate antagonist, inhibits T cell proliferation and the production of proinflammatory cytokines. In a clinical trial where it was used as monotherapy, methotrexate achieved disease stabilization and moderate repigmentation in most patients [75].

Regarding combination therapy, which integrates phototherapy with either topical or systemic treatments, meta-analytic evidence supports its superior efficacy over monotherapy. Notably, the combination of phototherapy with antioxidants, topical corticosteroids, or calcineurin inhibitors resulted in higher repigmentation rates and shorter treatment durations [76]. Phototherapy, particularly NB-UVB and excimer laser, promotes melanogenesis and melanocyte renewal, and its therapeutic effects are enhanced when used as part of a multimodal approach [77]. A summary of emerging therapies and clinical findings in vitiligo treatment is presented in Table 6.

## 5. Discussion

The treatment and management of vitiligo have long posed a significant challenge for dermatologists, with many existing therapies lacking specificity. However, recent advancements in understanding the pathophysiological mechanisms of vitiligo have paved the way for more targeted, effective, and safer treatment options for patients [40].

The discovery of the JAK-IFNγ pathway as a key target in immune response modulation presents a promising strategy for effective vitiligo management. However, the complexity of the disease necessitates a careful approach, emphasizing the need for complementary therapies that balance efficacy and long-term safety [32]. The treatment landscape encompasses a wide range of therapeutic approaches, including topical and systemic corticosteroids, advanced phototherapy, and surgical interventions. Additionally, emerging strategies focus on melanocyte regeneration and immunomodulation, offering new avenues for more effective management [78].

The approval of topical ruxolitinib marked a turning point in vitiligo therapy, representing the first treatment specifically developed for this indication based on a molecular understanding of disease pathogenesis. Several oral JAK inhibitors, including ritlecitinib (JAK3/TEC), upadacitinib (JAK1), and povorcitinib (JAK1), are currently in phase 3 clinical trials and may soon provide systemic options for patients with extensive disease. However, JAK inhibitors alone do not eliminate tissue-resident memory T cells (Trm), which persist in previously affected skin and drive disease relapse upon treatment discontinuation. Anti-IL-15 biologics targeting Trm survival represent a potentially disease-modifying approach that warrants further investigation. Combination strategies integrating immunosuppression (JAK inhibitors or calcineurin inhibitors) with melanocyte-stimulating modalities (NB-UVB phototherapy, afamelanotide) have demonstrated synergistic efficacy in multiple studies, and are likely to become the standard of care as the evidence base matures.

Key priorities for future research include: prospective validation of combination regimens in adequately powered randomized controlled trials; development of predictive biomarkers (such as serum CXCL10) to guide treatment selection; long-term safety assessment of systemic JAK inhibitors in the vitiligo population [79]; clinical advancement of anti-IL-15 biologics through pivotal trials; and exploration of novel drug delivery systems to improve therapeutic response in treatment-resistant anatomical sites such as acral areas [80].

This review has several limitations. As a narrative review, the literature search was not performed according to a systematic protocol (e.g., PRISMA), and selection bias cannot be excluded. The rapid pace of drug development means that some trials registered during the preparation of this manuscript may not be included. Furthermore, the heterogeneity of study designs, outcome measures, and patient populations among the cited studies limits direct comparison of therapeutic efficacy across different modalities. Finally, this review focuses primarily on pharmacological and phototherapeutic interventions and does not comprehensively address the psychosocial aspects of vitiligo or health economic considerations, which are important for holistic disease management.

## 6. Conclusions

Vitiligo management is shifting from non-specific immunosuppression toward mechanism-based therapy. Topical ruxolitinib and emerging oral JAK inhibitors, combined with phototherapy and melanocyte-directed approaches, offer improved and more durable repigmentation, while strategies targeting tissue-resident memory T cells may help address relapse. Individualized, combination-based regimens supported by well-designed randomized controlled trials are likely to define the future standard of care.

## Figures and Tables

**Table 1:** Summary of vitiligo types, prevalence, clinical presentation and recommended management.

Type/Subtype	Frequency	Clinical Features	Standard Management	Source
Non-segmental vitiligo (NSV; vitiligo non-segmental)	85–90%	symmetrical patches; face/hands/legs; friction sites; progressive	TCS/TCI ± NB-UVB; follow-up/maintenance	Rosmarin et al. 2024 [4]; Bergqvist & Ezzedine, 2021 [1,5]; Prajapati et al. 2025 [5]
Universal vitiligo (NSV subtype)	1–9%	near-complete depigmentation of skin and hair	As with NSV	Mascarenhas et al. 2024 [6]; Mahajan et al. 2019 [7]; van Geel et al., 2023 [3]
Acrofacial vitiligo (NSV subtype)	9–10%	extremities and face; periocular/perioral areas	As with NSV	Mahajan et al. 2019 [7]; Mascarenhas et al. 2024 [6]; van Geel et al., 2023 [3,8]
Segmental vitiligo (SV)	5–30%	unilateral; dermatomal; often stable after progression	TCS/TCI ± NB-UVB; surgery if stable and refractory	Bergqvist & Ezzedine, 2021 [1]; van Geel et al., 2023 [3]
Mucosal vitiligo	2–3%	mucosae: oral cavity, nose, genital areas	TCI; surgery	Kar & Raj 2018 [9]; Mahajan et al. 2019 [7]

**Table 2:** Summary of clinical studies on topical therapies for vitiligo.

Authors, Year	Study Type	Treatment	Treatment Parameters	Number of Patients	Efficacy
Seneschal et al., 2021 [40]	Multicenter, randomized, double-blinded, vehicle-controlled study	0.1% tacrolimus ointment	twice daily	42 (20 in tacrolimus group and 22 in vehicle group)	Therapeutic success in 65% of tacrolimus treated patients, relapse in 40% at 48 weeks
Lepe et al., 2003 [31]	Randomized double-blind trial (pediatric)	0.1% tacrolimus ointment vs. 0.05% clobetasol propionate	twice daily	20	Repigmentation: 49.3% (clobetasol) vs. 41.3% (tacrolimus); mild local reactions only
Kang, 2024 [36]	Drug review (Adis evaluation)	Ruxolitinib 1.5% cream (JAK1/2 inhibitor)	twice daily	Phase 2: 157; phase 3 (TRuE-V1/V2): 674	Significant facial and total-body repigmentation vs. vehicle; efficacy sustained up to week 104; mild local adverse effects (acne, pruritus, exfoliation)
Lo et al., 2010 [41]	Multicenter, open-label, non-comparative study	0.1% of tacrolimus ointment	twice daily	61 with 56 of patients being in follow-up for at least 4 weeks (sufficiently for estimating efficacy) and all patients having face and neck vitiligo	Repigmentation in all patients, 45.9% of patients showed at least mild repigmentation
Rosmarin et al., 2022 [38]	Two phase 3, double-blind, vehicle-controlled trials	1.5% ruxolitinib cream	twice daily	674 (221 treated with ruxolitinib and 109 in vehicle group in TRuE-V1 trial; 228 treated with ruxolitinib and 115 in vehicle group in TRuE-V2 trial)	More prominent repigmentation than vehicle group at week 24; 29.8% of patients in TruE-V1 and 30.9% of patients in TruE-V2 treated with ruxolitinib responded with a decrease of at least 75% from baseline in F-VASI (F-VASI75) at week 24

**Table 3:** Summary of phototherapy studies and findings in vitiligo treatment.

Authors, Year	Study Type	Treatment	Dosage	Number of Patients	Efficacy
Sapam et al., 2012 [48]	Randomized controlled trial	Narrowband UVB vs. oral PUVA	3 sessions/week for 6 months	53	Median repigmentation: 45% (NB-UVB) vs. 40% (PUVA); NBUVB had better tolerance and color match, fewer side effects
Bhatnagar et al., 2007 [8]	Open prospective comparative study	Narrowband UVB vs. PUVA	3 sessions/week up to 12 months	50	NBUVB showed higher stability (VIDA score) and greater efficacy than PUVA; stability achieved in 80% (NB-UVB) vs. 40% (PUVA).
Yones et al., 2007 [25]	Randomized double-blind trial	Narrowband UVB vs. oral PUVA	Twice weekly, up to 97 sessions	50	>50% repigmentation: 64% (NB-UVB) vs. 36% (PUVA); excellent color match in all NB-UVB patients; fewer adverse effects
Raone et al., 2018 [26]	Retrospective cohort study (15-year experience)	Narrowband UV-B (NB-UVB) phototherapy	310–315 nm, 2–3 times weekly	375	19 non-melanoma skin cancers (2.1%) diagnosed after mean of 5.2 years; no melanoma observed; increased risk of BCC and SCC mainly in elderly and psoriatic patients
Bhatnagar et al., 2007 [8]	Randomized open prospective study	Systemic PUVA vs. NB-UVB	NB-UVB 3×/week; PUVA 3×/week (TMP 0.6 mg/kg + UVA)	50 (25 NB-UVB/25 PUVA)	Mean repigmentation: NB-UVB 52.2% (6.3 months) vs. PUVA 44.7% (5.6 months); excluding hands/feet—NB-UVB 67.6% vs. PUVA 54.2% (*p* = 0.007)
Bakr et al., 2021 [43]	Prospective randomized comparative study	Fractional CO_2_ laser + tacrolimus/calcipotriol/NB-UVB	3 laser sessions 1 month apart + tacrolimus 0.03% 2× daily or calcipotriol 0.05% 2× daily or NB-UVB 2× weekly for 3 months	30 (10 per group)	Significant decrease in VASI score in all groups; highest repigmentation with laser + NB-UVB; no significant adverse events reported
Post et al., 2022 [46]	Systematic review/overview	Lasers (excimer, 308 nm, CO_2_, Q-switched) ± topical agents	Excimer laser 2–3× per week for ≥20 sessions; often combined with tacrolimus	meta-analysis including > 25,000 patients	Excimer laser ≈ NB-UVB in efficacy (≥75% repigmentation in ≈30% of patients); higher response with calcineurin inhibitors; CO_2_ laser useful for graft site preparation; good safety profile

**Table 4:** Summary of combined therapies studies and findings in vitiligo treatment.

Authors, Year	Study Type	Treatment	Dosage	Number of Patients	Efficacy
Lee et al., 2019 [51]	Systematic review and meta-analysis	Topical calcineurin inhibitors (tacrolimus, pimecrolimus) ± phototherapy (NB-UVB/excimer)	Tacrolimus 0.1% ointment or pimecrolimus 1% cream; applied twice daily; phototherapy 2–3×/week (median 3 months)	1499 (46 studies)	Monotherapy: ≥25% repigmentation 55%; ≥50% 38.5%; ≥75% 18.1%. Combination therapy: ≥25% 89.5%; ≥75% 47.5%. Better response on face/neck.
Utama et al., 2024 [39]	Scoping review	Janus kinase inhibitors (oral and topical: tofacitinib, ruxolitinib, baricitinib, ritlecitinib, upadacitinib, delgocitinib)	Oral or topical administration; often combined with phototherapy or other topical agents	35 studies reviewed (16 with oral tofacitinib; 6 with topical ruxolitinib; 6 with topical tofacitinib; smaller numbers for other JAKi)	JAK inhibitors showed promising repigmentation efficacy with favorable safety; mild acne and upper respiratory infections were the most common adverse effects; no serious hematologic or thromboembolic events reported; concurrent phototherapy improved outcomes.
Guyon et al., 2023 [53]	Retrospective case series	Systemic corticosteroid pulse + methotrexate ± phototherapy	Oral pulse steroids (weekend therapy or 3 days/month for 3 months) + MTX 10-15 mg/week	16	Mean VES of 13.13-6.66 (6 mo)-4.74 (12 mo); median VSAS of 6.5-1-0. Phototherapy accelerated repigmentation.
El Mofty et al., 2016 [54]	Randomized controlled trial	Oral mini-pulse steroid (OMP) ± NB-UVB	OMP (betamethasone/dexamethasone) 2 consecutive days/week; NB-UVB 2–3×/week for 3 months	45	NB-UVB ± OMP significantly more effective than OMP alone; both NB-UVB arms showed clinical and biochemical improvement (↑bFGF, ↓ICAM-1).
Chang & Sung, 2021 [55]	Systematic review and meta-analysis (RCTs)	Topical tacrolimus + NB-UVB or excimer laser	Tacrolimus 0.1% twice daily; NB-UVB or excimer 2–3×/week	10 RCTs	Combination therapy was superior to phototherapy alone: ≥50% repigmentation RR 1.355 (95% CI 1.17-1.57); ≥75% RR 1.47 (95% CI 1.12–1.92).
Zhu et al., 2023 [60]	Systematic review and network meta-analysis (RCTs)	NB-UVB alone vs. combinations (NB-UVB + tacrolimus/5-FU/Er:YAG/microneedling, etc.)	NB-UVB standard 2–3×/week + topical/adjunctive therapies	28 studies/1194 participants	All combination regimens achieved higher ≥75% repigmentation than NB-UVB alone; NB-UVB + tacrolimus OR 2.54 (95% CI 1.30–4.94).

**Table 5:** Summary of surgical treatment studies and findings in vitiligo treatment.

Authors, Year	Study Type	Treatment	Treatment Parameters	Number of Patients	Efficacy
Ju et al., 2021 [62]	Systematic review and meta-analysis	Surgical interventions	Analysis of 117 studies	8776	≥90% repigmentation in 52.7%; ≥50% in 81%; best results with thin skin grafting (72%); surgery effective and safe for stable vitiligo; mild local adverse effects (pain, color mismatch, scarring)
Chandela et al., 2023 [63]	Prospective interventional study	Autologous non-cultured epidermal cell suspension (NCES) without NB-UVB	Donor-to-recipient ratio of 1:10; follow-up of 6 months	27 patients (59 patches)	Excellent repigmentation in 64.4%, good in 28.8%, fair in 3.4%, poor in 3.4%; 67% mean repigmented area; low complication rate
Shahbazi et al., 2023 [64]	Prospective clinical study	Autologous melanocyte–keratinocyte transplantation (non-cultured)	Intraepidermal injection of 100–400 × 10^3^ cells/cm^2^; follow-up of 6 months	39	Excellent in 12.8%, good in 36%, moderate–minimal in 51.2%; significant repigmentation trend (*p* < 0.05); no adverse events reported

**Table 6:** Summary of emerging therapies and clinical findings in vitiligo treatment.

Authors, Year	Study Type	Treatment	Treatment Parameters	Number of Patients	Efficacy
Rosmarin et al., 2020 [30]	Multicenter, randomized, double-blind, controlled phase 2 trial	Ruxolitinib cream (JAK1/JAK2 inhibitor)	1.5% cream once or twice daily for up to 52 weeks	157 (33 in 1.5% BID group, 30 in 1.5% QD group, 32 control)	F-VASI50 achieved by 45–50% of patients with ruxolitinib vs. 3% placebo at week 24; sustained repigmentation up to week 52; mild adverse events (pruritus, acne at site)
Guttman-Yassky et al., 2024 [66]	Randomized, double-blind, placebo-controlled phase 2b biomarker substudy	Ritlecitinib (oral JAK3/TEC inhibitor)	10–200 mg QD (various loading doses) for 24 weeks	65	Dose-dependent F-VASI improvement at week 24; significant downregulation of pro-inflammatory biomarkers and upregulation of melanocyte markers (TYR, Melan-A); biomarker changes correlated with clinical response
Peeva et al., 2024 [68]	Post hoc analysis of phase 2b RCT	Ritlecitinib (oral JAK3/TEC inhibitor)	50 mg daily ± 4-week loading (100–200 mg) for 24 weeks	364 (247 light-skin; 117 dark-skin)	F-VASI improvement: −15.2 (light skin) and −37.4 (dark skin) vs. placebo (*p* = 0.004 and <0.0001); continued repigmentation up to week 48; similar safety across skin types
Neinaa et al., 2023 [69]	Randomized, double-blind, controlled comparative trial	Prostaglandin E2 vs. prostaglandin F2α (both with NB-UVB)	Intradermal injection weekly × 12 weeks + NB-UVB twice weekly × 3 months	30	Both PGE2 and PGF2α + NB-UVB significantly superior to NB-UVB alone; PGE2 induced faster onset and higher patient satisfaction; no side effects reported
Mutalik & Rasal, 2024 [70]	Technical innovation/case experience	Non-cultured autologous melanocyte–keratinocyte cell transplantation (NCMKCT) using an inexpensive autoclaved-box incubator	Split-thickness graft soaked in 0.25% trypsin; incubated 45–60 min in sterilized steel box at 32–40 °C	Not applicable	Consistent good repigmentation achieved in all treated patients; modification effective and low-cost; no adverse effects reported
Nuntawisuttiwong et al., 2024 [71]	Retrospective observational study (7-year data)	Autologous non-cultured melanocyte–keratinocyte transplantation procedure (MKTP)	One-day procedure; follow-up > 12 months	23 patients/24 lesions	Long-term sustained repigmentation (~80–90%); safe and effective
Bishnoi et al., 2024 [74]	Narrative review	Janus kinase inhibitors (topical ruxolitinib 1.5% cream, oral tofacitinib) + phototherapy (NB-UVB, excimer laser)	Topical ruxolitinib 1.5% BID for up to 52 weeks; oral tofacitinib dosing varies in studies	Not applicable	JAK inhibitors show high repigmentation rates with mild local AEs
Jafarzadeh et al., 2024 [75]	Systematic review of clinical trials and case series (2010–2023)	Systemic emerging therapies: JAK inhibitors (tofacitinib, baricitinib), afamelanotide, apremilast, methotrexate, cyclosporine, OMP steroids, antioxidants, etc.	Varies by included study (oral or injectable regimens)	2413 across 42 studies	Multiple systemic options effective; best evidence for JAK inhibitors, afamelanotide, apremilast, and methotrexate; minimal safety concerns
Luo et al., 2025 [76]	Network meta-analysis of RCTs	Phototherapy + topical combinations (NB-UVB or excimer with antioxidants, corticosteroids, etc.)	Per trial protocols (NB-UVB/EL plus topical agent)	27 trials, 2417 lesions (patches)	Phototherapy + antioxidants ranked highest for ≥50% and ≥75% repigmentation

## Data Availability

No new data were created or analyzed in this study.

## Abbreviations

The following abbreviations are used in this manuscript:

TCS	Topical corticosteroid
TCIs	Topical calcineurin inhibitors
NB-UVB	Narrowband ultraviolet B
NSV	Nonsegmental vitiligo
SV	Segmental vitiligo
ROS	Reactive oxygen species
DAMP	Damage-associated molecular pattern
IFN-γ	Interferon-gamma
CXCL	C-X-C motif chemokine ligand
JAK	Janus kinase
STAT	Signal transducer and activator of transcription
MHC	Major histocompatibility complex
HLA	Human leukocyte antigen
GWAS	Genome-wide association study
SASP	Senescence-associated secretory phenotype
MMP	Matrix metalloproteinase
Trm	Tissue-resident memory T cell
Treg	Regulatory T cell
NK	Natural killer (cell)
ILC	Innate lymphoid cell
FDA	U.S. Food and Drug Administration
EMA	European Medicines Agency
PUVA	Psoralen plus ultraviolet A
VIDA	Vitiligo Disease Activity Score
VASI	Vitiligo Area Scoring Index
F-VASI	Facial Vitiligo Area Scoring Index
VES	Vitiligo Extent Score
VSAS	Vitiligo Signs of Activity Score
NMSC	Non-melanoma skin cancer
BCC	Basal cell carcinoma
SCC	Squamous cell carcinoma
OMP	Oral mini-pulse therapy
MTX	Methotrexate
MPG	Mini/micro-punch graft
STSG	Split-thickness skin graft
NCES	Non-cultured epidermal cell suspension
MKTP	Melanocyte–keratinocyte transplantation procedure
PGE2	Prostaglandin E2
PGF2α	Prostaglandin F2 alpha
α-MSH	Alpha-melanocyte-stimulating hormone
